# Global production capacity of seasonal and pandemic influenza vaccines in 2019

**DOI:** 10.1016/j.vaccine.2020.12.018

**Published:** 2021-01-15

**Authors:** Erin Sparrow, James G Wood, Christopher Chadwick, Anthony T. Newall, Siranda Torvaldsen, Ann Moen, Guido Torelli

**Affiliations:** a The World Health Organization, Geneva, Switzerland; b School of Public Health and Community Medicine, UNSW Sydney, NSW, Australia; c Institute of Global Health, Faculty of Medicine, University of Geneva, Switzerland; d Women and Babies Research, The University of Sydney Northern Clinical School, NSW, Australia

**Keywords:** Vaccine, Influenza, Seasonal, Pandemic, Production, Capacity, CVV, candidate vaccine virus, ERL, Essential Regulatory Laboratories, GAP, Global Action Plan for Influenza Vaccines, GISRS, Global Influenza Surveillance and Response System, HA, haemagglutinin, IIV, inactivated influenza vaccine, LAIV, live attenuated influenza vaccine, LMIC, Low- and middle-income countries, NRA, national regulatory authority, PIP, Pandemic Influenza Preparedness Framework, QIV, quadrivalent influenza vaccine, SMTA2, Standard Material Transfer Agreements 2, TIV, trivalent influenza vaccine, VLP, virus-like particle

## Abstract

Vaccines will be an important element in mitigating the impact of an influenza pandemic. While research towards developing universal influenza vaccines is ongoing, the current strategy for vaccine supply in a pandemic relies on seasonal influenza vaccine production to be switched over to pandemic vaccines. Understanding how much vaccine could be produced, in which regions of the world and in what timeframe is critical to informing influenza pandemic preparedness. Through the Global Action Plan for Influenza Vaccines, 2006–2016, WHO promoted an increase in vaccine production capacity and monitors the landscape through periodically surveying influenza vaccine manufacturers. This study compares global capacity for production of influenza vaccines in 2019 with estimates from previous surveys; provides an overview of countries with established production facilities; presents vaccine production by type and manufacturing process; and discusses limitations to these estimates. Results of the current survey show that estimated annual seasonal influenza vaccine production capacity changed little since 2015 increasing from 1.47 billion to 1.48 billion doses with potential maximum annual influenza pandemic vaccine production capacity increasing from 6.37 billion to 8.31 billion doses. However, this figure should be interpreted with caution as it presents a best-case scenario with several assumptions which may impact supply. Further, pandemic vaccines would not be immediately available and could take four to six months for first supplies with several more months needed to reach maximum capacity. A moderate-case scenario is also presented of 4.15 billion doses of pandemic vaccine in 12 months. It is important to note that two doses of pandemic vaccine are likely to be required to elicit an adequate immune response. Continued efforts are needed to ensure the sustainability of this production and to conduct research for vaccines that are faster to produce and more broadly protective taking into account lessons learned from COVID-19 vaccine development.

## Highlights

• Annual seasonal vaccine production capacity is estimated at 1.48 billion doses.
• Best-case annual production for pandemic vaccine is estimated at 8.31 billion doses.
• Modest growth in pandemic influenza vaccine production capacity since 2015.
• The majority of production is occurring in High Income Countries.
• Challenges remain regarding maintenance of capacity and equitable distribution.

## 1 Introduction

The year 2018 marked 100 years since the onset of “Spanish flu” which spread around the globe in three waves, estimated to have infected one third of the global population and to have killed up to 50 million people [1], [2]. At that time, neither a vaccine nor conventional antibiotics to treat secondary bacterial infections were available and the causative agent remained unknown for many years [3], [4], [5]. It was not until the early 1930s that influenza A viruses were first isolated [6]. Since 1918, three less severe influenza pandemics have occurred: A(H2N2) in 1957 “Asian influenza”, A(H3N2) in 1968 “Hong Kong Influenza” and A(H1N1)pdm09 in 2009 [7], [8]. While the estimated attributable excess mortality due to the last three pandemics was far less than that of the 1918 pandemic, the threat of a severe pandemic caused by a respiratory viral infection remains. This threat is now being demonstrated, not by influenza but through the ongoing COVID-19 pandemic, and the world is currently racing to develop a vaccine for a pathogen where no vaccine has ever existed. By using innovative trial design and accelerated regulatory pathways, some speculate that it may take as little as 12–18 months to develop a COVID-19 vaccine while others feel this is too optimistic, and questions are being raised about whether the scale of production will be able to meet global needs [9], [10]. As of November 2020, eight months after the pandemic was declared, several vaccine candidates have reached clinical trials with some showing very promising preliminary efficacy results [11], [12], [13].

When it comes to pandemic influenza vaccines, while the timeframes are still not optimal, the world is better prepared with over 70 years of influenza vaccine research, production and use [3]. Coupled with infection prevention and control and clinical case management, vaccination will play an important role in mitigating the spread and impact of an influenza pandemic. An effective and safe vaccine provides a way to potentially ease socially and economically restrictive public health measures, including physical distancing, lock-downs and travel bans. However, in order to protect the global population, sufficient doses of vaccine will be required and will need to be distributed in a timely, effective and equitable manner.

As influenza evolution is a complex process, involving mutation and recombination as well as multiple hosts, it is not currently possible to predict when the next influenza pandemic will occur and what the exact strain will be. In practice this currently means that production of candidate vaccines will likely occur after a pandemic strain is identified and there will not be an immediate supply of vaccine. This also means that constant global surveillance of influenza viruses is required. Through the Global Influenza Surveillance and Response System (GISRS) circulating influenza viruses are closely monitored and assessed for pandemic potential. Based on this risk assessment, WHO issues recommendations for the development of candidate vaccines viruses (CVVs) for zoonotic influenza subtypes with potential to cause a pandemic in humans; this is done in addition to the twice annual recommendations for the composition of seasonal influenza vaccines for the Northern and Southern Hemispheres [14]. In addition to having a readily-available stockpile of CVVs for these potentially pandemic viruses, further development of zoonotic influenza virus vaccine candidates, such as for H7N9 and H5N1 subtypes, may also prove beneficial as this provides manufacturing experience with these strains and can inform on production yields as well as dosing requirements based on immunogenicity assessment in preclinical studies and in clinical trials [15], [16], [17]. All of these steps can potentially save time in the race to produce and distribute a pandemic influenza vaccine.

Although new platform technologies and improved influenza vaccines are on the horizon, the current strategy for vaccine supply in a pandemic still relies heavily on seasonal influenza vaccine production capacity. Existing production facilities for seasonal influenza vaccines can be switched over to produce pandemic influenza vaccines that could potentially become available to the population between four and six months after the declaration of a pandemic [18], [19]. There are currently three types of seasonal vaccines available on the market, inactivated influenza vaccines (IIV), live attenuated influenza vaccines (LAIV) and recombinant vaccines [20]. Current influenza vaccines can be manufactured using embryonated chicken eggs (egg-based) or using cell-culture (cell-based) as the substrate for production [21]. Understanding the types and amount of vaccine that could be produced on a global scale, in what timeframe and under which conditions is a critical component for planning the response to an influenza pandemic. This knowledge can also provide guidance on the role that novel platform technologies, such as mRNA vaccines, could play in overcoming such limitations or in augmenting supply.

In 2006, WHO launched the Global Action Plan for Influenza Vaccines (GAP) to serve as a ten-year strategy with the overarching goal to increase equitable access to pandemic influenza vaccines, including through increasing global production capacity to be able to produce enough vaccine to immunize 70% of the world’s population with two doses of a pandemic vaccine within six months from the availability of the vaccine virus strain to manufacturers. While one dose of vaccine was sufficient to confer adequate immune protection during the 2009 A(H1N1)pdm09 pandemic, a two-dose strategy may be needed for other influenza subtypes, such as H5N1, to which the wider population has no prior immunity. At the launch of the GAP, the annual global production capacity for influenza vaccines was estimated to be just 500 million doses of seasonal vaccine and 1.5 billion doses of pandemic vaccine with the vast majority of production located in high-income countries [22]. Ten years later, at the close of the GAP, this annual production capacity was estimated to have almost tripled, reaching 1.47 billion doses of seasonal vaccine and potentially translating to capacity to produce 6.37 billion doses of monovalent pandemic vaccine [22]. In addition, an expansion of production capacity in low- and middle-income countries (LMICs) was observed [22]. This expansion in production capacity was partially due to a technology transfer project under GAP where WHO, supported by partners including US Biomedical Advanced Research and Development Authority (BARDA) and PATH, provided seed funding and technical support to vaccine manufacturers located in LMICs to establish local influenza vaccine manufacturing [23].

However, this capacity was still insufficient to meet the GAP targets of two doses for 70% of the population and the production was estimated over a 12-month period. The estimate on pandemic vaccine production also represented a best-case scenario with several assumptions and limitations [22].

Moreover, to improve equitable access to pandemic influenza vaccines, under the Pandemic Influenza Preparedness (PIP) Framework, WHO has entered into legally binding Standard Material Transfer Agreements 2 (SMTA2) with industry to ensure manufacturers’ commitment to providing vaccines (and other products) to WHO in the event of an influenza pandemic to distribute to countries in need. Under these agreements, vaccine manufacturers commit to donate a percentage of their real-time vaccine production to WHO and reserve a percentage of real-time vaccine production at affordable pricing to WHO. As of May 2020, 13 influenza vaccine manufacturers have signed SMTA2 agreements [24].

In February 2019, building on the global assets and capacities established through GISRS, GAP and the PIP Framework, WHO launched the Global Influenza Strategy 2019–2030 to provide an overarching mechanism for WHO, countries and other stakeholders to strengthen influenza prevention, control and preparedness [25]. To assess implementation of the strategy, WHO will also continue to monitor global production capacity for influenza vaccines as one of six high level measures.

In this study we sought to update previous estimates [22], [26], [27] including stratifying the estimates by vaccine technology and by World Bank Income classification of countries [28]. We present a best-case scenario and a moderate case scenario. In addition, we sought to determine what amount of vaccine might be available to WHO for distribution to countries in need through the PIP SMTA2 mechanism. The results of this study also provide a baseline figure for the monitoring of vaccine production capacity under the Global Influenza Strategy. This article summarizes the results of a review of influenza vaccine manufacturing and a survey administered to influenza vaccine manufacturers to assess their production capacity for seasonal influenza and pandemic influenza vaccines.

## 2 Methods

### 2.1 Methods for gathering information

WHO maintains a database of influenza vaccine manufacturers, including information on vaccine type, formulation and substrate for production. Prior to issuing this survey, in April 2019, a desk review of manufacturers’ websites and a Google search using the manufacturers’ names and “influenza” was conducted to determine if any major changes had been reported since the 2015 survey as described in news articles or press releases. Reviews of PubMed and clinicaltrials.gov were also conducted in May 2019 to determine if any phase 3 seasonal trials had taken place since 2015 with influenza vaccines from new producers. The PubMed review used keywords “Influenza[Title] AND vaccine[Title] AND 2015/01/01:2019/05/01[dp]” and was filtered by Clinical Trial, Phase 3. The Clinicaltrials.gov search used keywords “influenza” under the field Condition or disease; “Vaccine” under other types and was limited to Interventional Studies (Clinical Trials) and Phase 3, the search was limited to studies starting from 1 January 2015 to 1 May 2019. The websites of new producers were searched to see if the product development was still active and if so they were then contacted to see if they had completed product development before sending the survey.

A series of questions (see supplementary material) was developed in Microsoft Word and the survey was sent to 36 manufacturers via email. Manufacturers were informed that all collected data would be presented in an aggregated manner for the purpose of estimating global production capacity of influenza vaccines. The survey was based on previous surveys with additional questions added [22], [26], [27]. Questions included general information about the types of influenza vaccines being produced (production platform, formulation, etc.) and manufacturers were asked to report maximum production capacity if operating at full scale. Questions about access to dose sparing adjuvants and whether they expected to have adequate supplies and filling capacity to meet their maximum capacity in the event of a pandemic were included.

We included only established influenza vaccine manufacturers in our dataset. “Established” manufacturers were defined as those that had already achieved licensure of influenza vaccines and with a currently functional production facility or with licensed facilities on standby for the production of vaccine in the event of a pandemic (relevant for one manufacturer). We excluded manufacturers with facilities or vaccines still under development; however, these will be monitored and included in future surveys if successful in their product development and licensure.

For manufacturers that did not reply to our survey, provided that they still appeared to be active in influenza vaccine production as confirmed by their website or through their national regulatory authority (NRA), we used publicly available data or information shared with WHO previously [22] for which WHO has permission to use in an aggregated manner.

Triangulation of the data was done using several sources: this survey, previously reported information, and information in the public domain to ensure there we no major discrepancies.

### 2.2 Methods for calculating production capacity

For these estimates we calculated production capacity as number of doses able to be produced, rather than number of vaccine courses, which, in a pandemic, could be more than one dose per person. Seasonal influenza vaccines contain either three strains of influenza (trivalent influenza vaccines, TIV) or four strains (quadrivalent influenza vaccines, QIV). For the best case-scenario we assumed that the same amount of antigen normally needed for each seasonal strain would be sufficient for a monovalent pandemic vaccine and that the vaccine virus strain would be able to be produced at similar yields to seasonal viruses, therefore pandemic production capacity can be calculated by multiplying seasonal capacity by three (TIV) or by four (QIV). For the moderate case scenario we assumed that twice the amount of antigen would be required to elicit an adequate immune response. We applied this method to calculate potential monovalent pandemic vaccine capacity based on a manufacturer’s seasonal production capacity unless a manufacturer provided another figure based on their own assessments. We also factored in adjuvants for some manufacturers as described below.

Some adjuvants have been shown to enhance the immune response and have dose sparing effects for both inactivated and recombinant influenza vaccines. While several adjuvants are in development for influenza vaccines, the adjuvants that we factored into our estimates were MF59 and AS03. Both adjuvants are squalene-based, have been demonstrated to have dose-sparing effects in randomized controlled clinical trials and are also present in several licensed influenza vaccines [29]. Their dose sparing effects have varied depending on the subtype of influenza. For H5N1 and H7N9 inactivated influenza vaccines that have been tested in clinical trials, the dose sparing effects of these adjuvants have allowed an adequate immune response at either 3.75 µg of haemagglutinin (HA) (a quarter of the amount of antigen present for each strain of seasonal influenza) or 7.5 µg of HA (half the amount of antigen present for each strain of seasonal influenza) [30], [31], [32]. MF59 is used in FLUAD Pediatric™/FLUAD®, a seasonal inactivated influenza vaccine indicated for young children (6 months to less than 2 years of age) and older adults (65 years of age and older) as well as in a licensed H5N1 vaccine which contains 7.5 µg of H5N1 HA with two doses of the vaccine to be given 21 days apart [33], [34]. In the case of AS03 it has been included in licensed H5N1 and H1N1 vaccines at 3.75 µg of HA per dose [35], [36]. One dose was adequate for H1N1; for H5N1 two doses of vaccine given 21 days apart are required. We therefore factored in the increased capacity that might be achieved for three manufacturers with access to these adjuvants.

### 2.3 Assumptions used for calculating production capacity

There are several challenges that will impact the production of influenza vaccines in the event of a pandemic and several assumptions have been made in calculating this production capacity:

• Manufacturers would not be in the middle of seasonal vaccine production and could “switch” their production to pandemic vaccine immediately upon availability of the pandemic virus sequence or candidate vaccine virus.
• The pandemic influenza vaccine strain will grow equally well in eggs and cells as compared to seasonal vaccine viruses. While this was the case for H1N1, other subtypes may not always grow as well [17].
• In the event of a pandemic, there will be an adequate supply of eggs. That is, egg-laying poultry would not be compromised if the pandemic also affects poultry and adequate supplies would be available even if the pandemic occurs off season when some manufacturers may not normally order eggs.
• There would be no delays in any of the production steps. Influenza vaccine production is a time-intensive process, activities are often inter-related and a delay in one activity may impact the overall timelines.
• Manufacturers would have sufficient filling lines available to fill their vaccine into vials or syringes to meet their maximum capacity.
• Adequate workforce protection measures would be in place to ensure continued production.
• There would be a sufficient and timely supply of other reagents and supplies, such as vials, syringes and functioning transport networks, to deliver the vaccine.
• Adjuvants (MF59 and AS03) would allow half the amount of antigen to result in an adequate immune response, thus doubling capacity for those manufactures with access to these adjuvants.

## 3 Results and discussion

In order to maintain individual manufacturer confidentiality production capacity figures are presented in an aggregated manner.

### 3.1 Production landscape

Of the 36 manufacturers identified for the survey, three were found to be only conducting fill/finish and packaging operations, and two replied that they were no longer producing influenza vaccines. These five were excluded from our analysis. Seven did not complete the survey but responded to confirm that they were still active and provided information on their production capacity. Five manufacturers did not reply but still appeared active (as confirmed through their website or in some cases by the NRA), in this case we used information reported previously to WHO [22] and supplemented this with any new information found in the public domain. For one new manufacturer with a product licensed in 2019 the information on production capacity was obtained from the NRA. In total 18 manufacturers completed the 2019 survey.

**Fig. 1:**
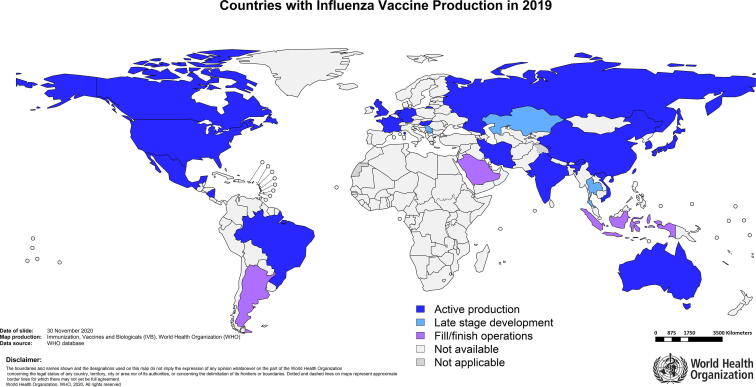
Countries with influenza vaccine production and fill/finish facilities in 2019 (active or in late stage development).

A total of 31 national and multinational vaccine manufacturers with a combined total of 40 different influenza vaccine bulk manufacturing facilities in 18 countries are included in our final dataset (Table 1). Countries with at least one active influenza vaccine production facility are: Australia, Brazil, Canada, China, France, Germany, Hungary, India, Iran (Islamic Republic of), Japan, Mexico, Nicaragua, Russian Federation, Republic of Korea, the Netherlands, Viet Nam, United Kingdom of Great Britain and Northern Ireland and United States of America. It should be noted that manufacturers in an additional three countries, Kazakhstan, Serbia and Thailand, were in the final stages of establishing influenza vaccine production capacity in 2019 (see Fig. 1). We also found that fill/finish operations for influenza vaccines are taking place in Argentina, Indonesia and Saudi Arabia, although we did not do a comprehensive review of fill/finish facilities. Production is occurring in almost all regions of the world with the exception of Africa (see Table 2). It is important to note that of the 40 active production facilities, none are located in low-income countries, five are in lower-middle income countries, 15 in upper-middle income and 20 in high-income countries (see Table 3).

**Table 1:** Established influenza vaccine manufacturers.*

Manufacturer	Bulk vaccine production sites (countries)	Vaccine type
Abbott Biologicals B.V	The Netherlands	IIV, egg-based
Adimmune Corporation	China	IIV, egg-based
AstraZeneca PLC	The United Kingdom	LAIV, egg-based
Bayerpaul Group	Iran (Islamic Republic of)	IIV, egg-based
BIKEN Co., Ltd	Japan	IIV, egg-based
Changchun BCHT Biotechnology Co.	China	LAIV, egg-based
China National Biotec Group (CNBG)	China (2 facilities)	IIV, egg-based
CPL biologicals Pvt. Ltd	India	recombinant VLP, cell-based
Daiichi Sankyo	Japan	IIV, egg-based for seasonal, cell-based for pandemic
Dalian Aleph Biomedical Co., Ltd.	China	IIV, egg-based
Denka Seiken Co., Ltd.	Japan	IIV, egg-based
Fluart Innovative Vaccines Kft	Hungary	IIV, egg-based
FORT, ltd.	Russian Federation	IIV, egg-based
GC Pharma	Republic of Korea	IIV, egg-based
GlaxoSmithKline (GSK)	Canada, Germany	IIV, egg-based
Hualan Biological Engineering Inc.	China	IIV, egg-based
Il-Yang Pharm	Republic of Korea	IIV, egg-based
Instituto Butantan	Brazil	IIV, egg-based
Institute of Vaccines and Medical Biologicals (IVAC)	Viet Nam	IIV, egg-based
Jiangsu GDK Biotechnology Co., Ltd.	China	IIV, egg-based
KM Biologics Co., Ltd.	Japan	IIV, egg-based for seasonal, cell-based for pandemic
Mechnikov Institute	Nicaragua	IIV, egg-based
Microgen	Russian Federation	IIV and LAIV, egg-based
Sanofi Pasteur	China, France, Japan, Mexico, the USA (2 facilities)	IIV, egg-based and recombinant, cell-based
Seqirus	Australia, the United Kingdom, the USA	IIV, egg-based and cell-based
Serum Institute of India Pvt. Ltd.	India	LAIV, egg-based
Sinovac Biotech Ltd.	China	IIV, egg-based
SK Bioscience	Republic of Korea	IIV, cell-based
SPbNIIVS	Russian Federation	IIV, egg-based
Takeda	Japan	Approved facility for cell-based pandemic IIV
Zydus Cadila	India	IIV, egg based

* Information presented in this table is available in the public domain as provided in supplementary information Table 1.

**Table 2:** Number of active influenza vaccine production facilities by WHO region.

WHO Region	Number of production facilities
African Region	0
Region of the Americas	7
Eastern Mediterranean Region	1
European Region	9
South-East Asia Region	3
Western Pacific Region	20

**Table 3:** Number of active influenza vaccine production facilities by income status of country of production.*

Income status	Number of production facilities	% of global production capacity (seasonal)	% of global production capacity (pandemic)	% of world population**
Low-income	0	0%	0%	9%
Lower-middle income	5	2%	1%	38%
Upper-middle income	15	29%	19%	37%
High-income	20	69%	80%	16%

* World Bank Classification 2019.

** World Bank Population 2019, https://databank.worldbank.org/data/download/POP.pdf.

### 3.2 Production capacities

Estimated production capacity is described below and summarized in Table 4.

**Table 4:** Summary of estimated production capacities in 2019.

Breakdown of production capacities	Seasonal Influenza	Pandemic influenza
**Total Annual Production Capacity**		

Seasonal influenza vaccines	1.48 billion doses	
Pandemic influenza vaccines (moderate case)		4.15 billion doses
Pandemic influenza vaccines (best case)		8.31 billion doses

**By vaccine type**		

IIV	89.6%	88.9%
LAIV	5.0%	3.4%
Recombinant	5.4%	7.7%

**By substrate**		

Embryonated eggs	84.5%	79%
Cell culture	15.5%	21%

#### 3.2.1 Seasonal influenza vaccines

**Fig. 2:**
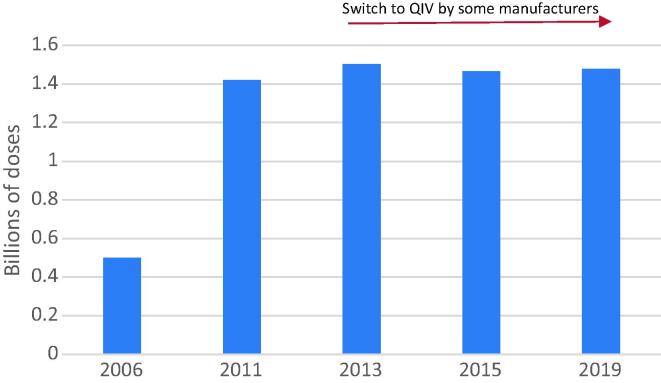
Estimated total annual seasonal production capacity over time (since 2006).

On a global scale, in 2019, there was estimated capacity to produce 1.48 billion doses of seasonal influenza vaccines over a 12-month period (Fig. 2). It is important to note that this is not an estimate of current production, which is based on demand for seasonal influenza vaccines, but rather how much vaccine could be produced if manufacturers were to operate at full scale. While seasonal influenza vaccine production capacity appears to have stabilized since 2011 in terms of number of doses, this does not take into account increasing production of QIV (containing an additional B strain) by several large-scale manufacturers since its introduction in 2013, requiring typically an extra 33% of antigen per dose. This means that maximum production capacity for monovalent pandemic vaccines has continued to increase over this period despite the levelling off in production of individual seasonal doses. It should also be noted that six small scale facilities ceased production of influenza vaccines since the last survey in 2015, and, while this has been offset by the establishment of seven new producers, this is of concern as it demonstrates issues with the sustainability of smaller producers. A few additional producers are expected to come online in the coming years and will be included in potential future surveys. Demand for seasonal influenza vaccines has remained level in recent years [37], [38], although there have been reports of increasing demand for influenza vaccines in 2020 related to the current COVID-19 pandemic and concerns about co-circulation with influenza [39], [40], [41].

The majority of seasonal vaccines are produced using embryonated eggs, representing 84.5% of global production capacity while cell-based vaccines represent 15.5% of capacity. Inactivated influenza vaccines (IIV) represent 89.6% of global production capacity for seasonal influenza vaccines. In general, seasonal IIV are dosed at 15 µg of antigen per strain, meaning that in total seasonal vaccines contain either 45 µg of total antigen for TIV, which cover three influenza strains, or 60 µg of antigen for quadrivalent vaccines QIV, which cover four strains. IIV are the most widely used influenza vaccines and can be given to all individuals from 6 months of age [42].

For live attenuated influenza vaccines (LAIV), there are two types available on the market, based on either the “Ann Arbor” backbone or the “Leningrad” backbone. Potency, or antigen content for seasonal vaccines is determined as 6.5–7.5 log fluorescent focus units (FFU) for Ann Arbor-based LAIV or at least 6.5–7 log 50% egg infected dose (EID_50_) for Leningrad-based LAIV [43], [44]. There is one producer of the Ann Abor LAIV (AstraZeneca PLC) and three producers of the Leningrad LAIV (Changchun BCHT Biotechnology Co., Microgen, Serum Institute of India Pvt. Ltd.). LAIV is generally recommended for use in children > 2 years and is contraindicated for pregnant women and immunocompromised individuals [42]. LAIV represent 5.0% of total seasonal influenza vaccine production capacity.

Regarding recombinant vaccines, there are only two types licensed: Sanofi Pasteur’s Flublok and CPL Biologicals’ Cadiflu-S. Flubok is a protein-based vaccine containing recombinant HA produced in insect cell culture using the baculovirus expression system. For seasonal influenza vaccines the HA content is 45 µg per antigen (or 180 µg for QIV) [45]. Cadiflu-S is produced by incorporating the gene sequences of three immunogenic proteins of influenza (HA, NA and M1) to produce a virus-like particle (VLP) vaccine using the baculovirus expression system [46]. Both vaccines are indicated for individuals above 18 years of age [47], [48]. Recombinant vaccines represent 5.4% of capacity.

Of the 30 manufacturers producing seasonal influenza vaccines, thirteen manufacturers produce TIV only, ten produce QIV only and seven manufacturers are producing both TIV and QIV.

#### 3.2.2 Pandemic influenza vaccines

**Fig. 3:**
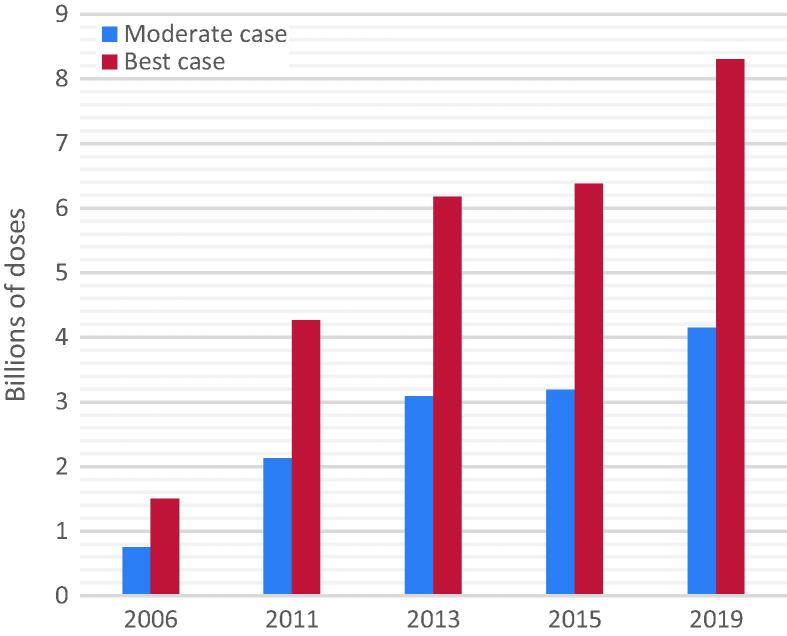
Estimated potential annual pandemic production capacity over time (since 2006). Best case scenario – manufacturers would be able to operate at full scale with no limitations on supplies/reagents, the pandemic strain would grow equally well in eggs/cells as seasonal strains and the same amount of antigen as normally used for each seasonal strain would be enough to elicit an adequate immune response. Moderate case scenario – manufacturers would be able to operate at full scale with no limitations on supplies/reagents, the pandemic strain would grow equally well in eggs/cells however twice the amount of antigen as per each strain of seasonal would be required to elicit an adequate immune response.

We calculated that in a best-case scenario, an estimated 8.31 billion doses of pandemic vaccine could be produced in a 12-month period. Assuming a moderate-case scenario, we estimated that 4.15 billion doses could be produced (Fig. 3). However, there are many factors that may impact this production as described above in Section 2.3. Production capacity for pandemic vaccines has continued to grow since 2006, when it was estimated to be just 1.5 billion doses. Increased production facilities, a switch of many producers to quadrivalent vaccines, and access to dose-sparing adjuvants is a major contributing factor. Since the last survey in 2015, pandemic vaccine capacity has expanded by nearly 2 billion doses.

Egg based vaccine production represents 79% of pandemic production capacity and 21% is cell based. These proportions differ from seasonal vaccine production as: there are two manufacturers who produce seasonal vaccine on eggs but have approval to produce cell-based vaccines for pandemics; some of the manufacturers with cell-based production produce QIV thus quadrupling their capacity for pandemic; and additionally, there is one manufacturer that has an approved facility for production of cell based IIV in the event of a pandemic but does not produce seasonal influenza vaccines. IIV represent 88.9% of pandemic production capacity while LAIV and recombinant vaccines represent just 3.41% and 7.7% of capacity, respectively (Table 4).

**Fig. 4:**
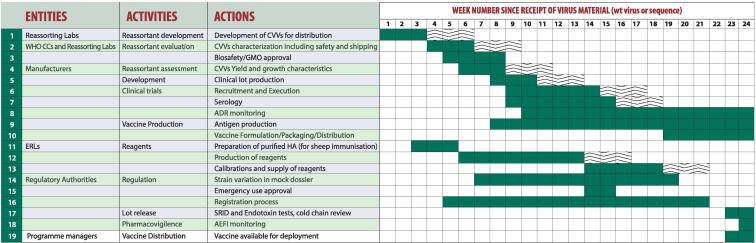
Timeline of pandemic vaccine production (IIV) [18].

Vaccine supply would not be immediate in the event of a pandemic and could take 4–6 months for first supplies of vaccine to be available to the population. It would then take several months of continued production to reach maximum capacity. For IIV, which represents 89% of pandemic vaccine production capacity, it is estimated that, if starting from scratch, it would take 23–24 weeks from the time that the recommendation of the pandemic virus is made and its genetic sequence uploaded to the subsequent availability of first doses of vaccine ready for deployment (see Fig. 4) [18], [49]. This timing takes into account: (1) the time needed for the CVV to be prepared, characterized, safety tested by the reassorting laboratories and shipped to manufacturers; (2) the time needed for the manufacturers to assess the CVVs for yield and growth characteristics, prepare clinical lots and conduct clinical trials and the time for vaccine production, formulation, packaging and distribution; (3) the time needed for the Essential Regulatory Laboratories (ERLs) to prepare reagents necessary to measure potency and for lot release; (4) the time for NRAs to assess the vaccine for licensure and release the vaccines [18].

The timing of production of LAIV is slightly shorter than inactivated vaccines, estimated to take 21 weeks from the time that the genetic sequence is uploaded to the subsequent availability of vaccine ready for deployment [18]. Recombinant vaccines are much faster to produce as there is no need to wait for CVVs to be developed [50]. However both LAIV and recombinant vaccines represent a small proportion of global production capacity.

It should also be noted that the timelines listed above represent ideal circumstances when all necessary inputs are in place (staff, facilities, manufacturing supplies, equipment) and function optimally and that a delay in one activity may have an impact on other activities. The timing also assumes that a production facility would not be in the middle of a campaign to produce seasonal influenza vaccine. Pandemic influenza vaccine response, including when to initiate production of the pandemic vaccine, is a complex process for a variety of reasons (e.g. manufacturers may have contractual obligations to fill orders of seasonal vaccines) [18]. Under the Global Influenza Strategy 2019–2030, WHO has committed to “develop or update guidance, including global vaccine preparedness plans for switching from seasonal to pandemic vaccine manufacturing” [25]. Additionally, WHO has outlined the overall pandemic influenza vaccine response processes within the Pandemic Influenza Risk Management Guidance and work is underway to further operationalize this guidance [51]. Bottlenecks in the manufacturing process have been identified and ongoing efforts are underway to shorten some of the timelines [49]. For example, a major bottleneck is the time needed to develop new reagents for the single radial immunodiffusion assay which is used to assess potency of influenza vaccines and efforts are ongoing to develop new potency assays for inactivated influenza vaccines [52], [53]. In addition, through GISRS, WHO periodically recommends the development of CVVs for zoonotic influenza viruses of pandemic potential. Such development can shave several weeks off timelines if a pandemic emerges from one of these strains (see Fig. 4). Further development of these candidate vaccines with producers provides manufacturing experience with these strains which leads to knowledge on production yields. Preclinical animal studies and clinical trials provide information on dosing requirements based on challenge studies, immunogenicity assessments as well as initial safety data for the vaccines. Such development is important for pandemic preparedness and has the potential to shorten timelines by 15 weeks. However, as demonstrated by the need to update seasonal vaccine strain composition on an annual basis, there can be great variance between viruses of the same influenza subtype and in some cases CVVs for zoonotic influenza viruses of pandemic potential developed previously have shown little cross-protectiveness to drifted viruses within the same subtype [54].

As shown in Table 3, the majority (80%) of production capacity for pandemic influenza vaccines is located in high-income countries, yet high-income countries represent only 16% of the global population. This highlights the need to ensure equitable distribution of vaccine to LMICs lacking in production capacity, which are often most at risk due to weak health systems and underlying medical conditions in their populations. Under the PIP framework commitments, we calculated that WHO has secured access to 11.3% of global pandemic influenza vaccine production for distribution to countries in need. While this amount would be insufficient to cover the entire population in LMICs, it would help to support vaccination of priority groups in countries where influenza vaccines are not being produced and in countries that do not have supply agreements in place with manufacturers. Given that vaccine will not become available until several months after the pandemic and initial supplies will be limited, during the first wave of the pandemic, identification of risk groups will be important to allow for rationalized use of limited vaccine supply. While the specific pandemic vaccine would still need to be prequalified by WHO in order to allow UN agencies to purchase or receive donations of the vaccine, as of May 2020, nine influenza vaccine manufacturers have prequalified at least one of their influenza vaccines representing 74% of pandemic vaccine production capacity. This is likely to help the prequalification of their pandemic vaccines.

The delay in availability of pandemic vaccine highlights the need to improve current production processes so that vaccines can be produced more rapidly and to develop new influenza vaccines that offer broader, longer lasting protection across different subtypes of influenza A viruses. Such vaccines could be used for seasonal influenza and stockpiled for use in a pandemic. WHO published guidance on preferred product characteristics for improved influenza vaccines in 2017 [55]. There are numerous groups and initiatives aimed towards developing universal or improved influenza vaccines. A recently launched initiative, the Universal Influenza Vaccine Technology Landscape, reported in April 2020 that 22 new influenza vaccine technologies had reached clinical trials and 74 were in late preclinical development [56].

### 3.3 Issues impacting manufacturers’ ability to maximize supply

With regards to issues that may impact a manufacturer’s ability to reach maximum capacity:

• Nine manufacturers out of 18 that responded to the question confirmed that they expect to have adequate access to eggs and ancillary supplies to achieve their maximum pandemic production capacity. Seven responded that access to eggs and supplies could be a limitation. Two replied that access to egg supply would be dependent on the time of year when the pandemic occurs (that is, if the pandemic were to occur when they do not normally source eggs for seasonal vaccine production). Supplementing with information provided in 2015 an additional five manufacturers stated that supplies would not be a bottleneck. Information was not available for eight manufacturers.
• Fourteen manufacturers out of 17 that responded to the question confirmed that they expect to have adequate access to filling lines to achieve their maximum production capacity. Three responded that filling capacity would be a limitation. Supplementing with data from 2015 an additional five manufacturers stated that filling capacity would not be a bottleneck. Information was not available for nine manufacturers.

The responses to these questions demonstrate that for some manufacturers, egg supplies and filling capacity could be major bottlenecks to achieving their maximum capacity. Additional fill/finish capacity at other facilities should be explored as well as options for sourcing or scaling egg supply.

## 4 Conclusions

There is a robust global platform for production of pandemic influenza vaccines based on seasonal influenza vaccine production facilities. There are 31 manufacturers with 40 production sites operating in 18 countries in almost all regions of the world with the exception of Africa. However, while LMICs represent 84% of the global population, 80% of pandemic influenza vaccine production capacity is located in high-income countries, with only 19% in upper-middle-income countries, 1% in lower-middle income countries and no production in low-income countries. One reason for this discrepancy may be the lack of demand for seasonal vaccines in many LMICs offering little or no internal market for local producers [57]. Underutilization of influenza vaccines in LMICs is likely due to many factors, including age demographics - with LMICs having in general younger populations, absence of burden of disease data and the high-cost associated with the need to re-vaccinate on an annual basis [58]. In the event of an influenza pandemic, vaccine production could be as high as an estimated 8.31 billion doses produced in 12 months; however, this assumes a best-case scenario for all manufacturers and in reality, is likely to be less. Two doses of vaccine may be required to elicit an adequate immune response, meaning that even in a best-case scenario there would only be enough vaccine to cover just over half of the world’s population. Furthermore, using current technologies and licensed facilities, first supplies of vaccine will likely not be available to the population until four to six months after the pandemic is declared. There are, however, efforts underway to shorten these timelines such as via additional development of CVVs for viruses with pandemic potential and these efforts could result in vaccine as early as 3 months.

Given that there is not likely to be vaccine available in the first wave of an influenza pandemic, controlling the pandemic initially will depend on strong basic public health measures as well as clinical management and the use of therapeutics including antivirals, monoclonal antibodies or other medicines to treat the host response. Vaccine will likely become available during the second wave, but initially supplies will probably be limited as production ramps up. It is important that risk-groups are identified during the first wave so that by the time that first supplies of vaccine are available vaccine distribution can be prioritized for these groups as well as other priority groups such as healthcare workers.

The original goal of the GAP was to produce enough vaccine to cover 70% of the global population with two doses of a pandemic vaccine (translating to 10.92 billion doses in 2019) within six months from the availability of the vaccine virus strain to manufacturers. While pandemic vaccine production capacity has considerably increased since that goal was set, growing from an estimated annual production of just 1.5 billion doses in 2006 to up to 8.31 billion doses in 2019, we are still 2 billion doses short of that target. Without increased demand for seasonal influenza vaccines, production is unlikely to grow significantly. In addition to enhanced surveillance of potentially pandemic strains and development of CVVs, continued efforts towards shortening current production timelines, expanding access to doses sparing adjuvants and for developing better, more broadly protective influenza vaccines are needed. Exploration of the role of novel platform technologies, such as mRNA vaccines as highlighted in the current COVID-19 pandemic, is also needed in addressing potential expansion of production capacity for influenza vaccines.

## Funding

This work was commissioned and supported by the World Health Organization.

## Disclaimer

The authors alone are responsible for the views expressed in this article, which do not necessarily represent the views, decisions or policies of the institutions with which the authors are affiliated.

## Declaration of Competing Interest

The authors declare that they have no known competing financial interests or personal relationships that could have appeared to influence the work reported in this paper.
