# Supplementary material for: Global production capacity of seasonal and pandemic influenza vaccines in 2019
Source: Vaccine. 2021 Jan 15;39(3):512–20. doi: 10.1016/j.vaccine.2020.12.018 (PMC7814984; doi:10.1016/j.vaccine.2020.12.018)
Supplement: Supplementary data 1 [file mmc1.docx]

**Estimating the current Global Production Capacity for Influenza Vaccines (2019)**

**Questionnaire to influenza vaccine manufacturers CONFIDENTIAL**

**Background:**

The World Health Organization (WHO) is contacting all known vaccine manufacturers with seasonal influenza vaccine production capacity in 2019. This follows on from similar surveys conducted in the past.^[[1]](#footnote-1)-4^ These periodic studies aim to estimate the global production capacity for pandemic influenza vaccines in order to contribute to measuring global preparedness for future pandemics.

The method used is to multiply **maximum** production capacity by 3 for those manufacturers producing trivalent vaccines and by 4 for quadrivalent vaccines. If a manufacturer has access to dose sparing adjuvants then this is also factored in. After completing the survey WHO will contact you to confirm our calculations for your maximum production capacity and for any clarifications.

The data provided to WHO will be used for the sole purpose of estimating global production capacity and will be presented in an aggregated manner in the published domain. For any use of data outside of this purpose you will be contacted separately to seek your consent.

**General Information:**

Company name and address:

**Questions:**

1. Which seasonal influenza vaccines do you produce:

Northern Hemisphere formulation

Southern Hemisphere formulation

Both Hemisphere formulations

1. Do you produce the bulk antigen (full production) or are you a fill/finish operation only:

Full production

Fill/finish only

Packaging only

1. If you are fill/finish or packaging only, from which manufacturer do you import the vaccine:

*(Note: if you are fill/finish/packaging only you do not need to complete further questions in this survey)*

1. In which countries is your vaccine licensed:

1. If your vaccine is not yet licensed, please indicate the expected date of first licensure:
2. If you do not already have a WHO prequalified (PQ) vaccine, have you considered applying for WHO PQ:

Vaccine is already prequalified

Yes

No

Please explain if needed:

1. Which type of influenza vaccine do you produce (check all that apply)

Inactivated

Live attenuated

Recombinant

Other

1. What is the valence of your vaccine:

Trivalent

Quadrivalent

Both

1. If you produce only trivalent, do you have plans to move to quadrivalent production in the near future:

Yes

No

Any comments:

1. Type of substrate used:

Egg

Cell

Both

1. If you produce on both eggs and cell, what proportion of your production is on each (in %)

Eggs:_______

Cells:_______

1. If you produce only on eggs, have you considered moving to cell-culture production:

Yes

No

Why/why not, please explain:

1. In which countries do you have production sites:

1. If you have more than one production site, please provide, if possible, the proportion of production occurring at each site (in %): __________________________________________________________________________________________________________________________________________________________________________________
2. What is your **maximum** production capacity in doses (if **operating at full scale**). *If you produce both quadrivalent and trivalent please list these separately*.

|  | Monovalent | Trivalent | Quadrivalent |
| --- | --- | --- | --- |
| 3 months |  |  |  |
| 6 months |  |  |  |
| 9 months |  |  |  |
| 12 months |  |  |  |

Comments:

1. How many monovalent doses can you produce per batch:
2. Please specify the time taken to produce one batch of monovalent bulk:
3. Do you have access to dose sparing adjuvants:

Yes

No

1. If yes, please specify the adjuvant:

1. Have you licenced any monovalent (pre-)pandemic influenza vaccines (e.g H5N1, H7N9)

Yes

No

If yes, please specify which ones:

1. Do you have Standard Operating Procedures or Agreements in place to source eggs and ancillary supplies if a pandemic occurs during your production off-season?

Yes

No

1. Do you expect to have adequate access to eggs and ancillary supplies to achieve your maximum production capacity if a pandemic occurs:

Yes

No

please explain if necessary:

________________________________________________________________________________________

1. In the event of a pandemic do you expect to have adequate access to filling lines to achieve your maximum production capacity:

☐Yes

☐No

please explain if necessary:

1. Do you have further comments to make with regards to your influenza vaccine production activities and capacity to respond to an influenza pandemic?

1. 1. Collin N, de Radiguès X. Vaccine production capacity for seasonal and pandemic (H1N1) 2009 influenza. *Vaccine* 2009; 27 (38): 5184–5186
   2. Partridge J, Kieny MP. Global production of seasonal and pandemic (H1N1) influenza vaccines in 2009–2010 and comparison with previous estimates and global action plan targets. *Vaccine* 2010; 28 (39): 4709–12.
   3. Partridge J, Kieny MP. Global production capacity of seasonal influenza vaccine in 2011. *Vaccine* 2013; 31(5): 728-31.
   4. McLean, Kenneth A. et al. “The 2015 Global Production Capacity of Seasonal and Pandemic Influenza Vaccine.” Vaccine 34.45 (2016): 5410–5413. PMC. Web. 10 May 2018.

   [↑](#footnote-ref-1)
