# Supplementary material for: Global production capacity of seasonal and pandemic influenza vaccines in 2019
Source: Vaccine. 2021 Jan 15;39(3):512–20. doi: 10.1016/j.vaccine.2020.12.018 (PMC7814984; doi:10.1016/j.vaccine.2020.12.018)
Supplement: Supplementary Table 1 [file mmc2.docx]

| **Manufacturer**  **Supplementary Table 1: Source of public information on manufacturers** |  | **Source of publicly available information** |
| --- | --- | --- |
| Abbott Biologicals B.V | <http://mri.cts-mrp.eu/download/NL_H_0137_001_FinalPL.pdf>;  <https://www.abbott.com/corpnewsroom/finance/abbott-brings-its-proven-flu-fighters.html>;  <https://www.medicines.org.uk/emc/files/pil.9381.pdf> | |
| Adimmune Corporation | [www.adimmune.com.tw/en/about_intro.php](http://www.adimmune.com.tw/en/about_intro.php);  [www.adimmune.com.tw/en/service_products.php](http://www.adimmune.com.tw/en/service_products.php) | |
| AstraZeneca PLC | [www.flumistquadrivalent.com/](http://www.flumistquadrivalent.com/);  [www.azpicentral.com/flumistquadrivalent/flumistquadrivalent.pdf](http://www.azpicentral.com/flumistquadrivalent/flumistquadrivalent.pdf);  <https://www.fda.gov/media/139946/download> | |
| Bayerpaul Group | [www.bayerpaul.com/en-Pages-204/VACCINES](http://www.bayerpaul.com/en-Pages-204/VACCINES) | |
| BIKEN Co., Ltd | [www.biken.or.jp/english/about_biken/products](http://www.biken.or.jp/english/about_biken/products);  [www.pmda.go.jp/files/000153640.pdf](http://www.pmda.go.jp/files/000153640.pdf) | |
| Changchun BCHT Biotechnology Co. | [www.bchtpharm.com/100039/827.html](http://www.bchtpharm.com/100039/827.html) | |
| China National Biotec Group (CNBG) | <https://www.cnbg.com.cn/>;  <https://www.cnbg.com.cn/content/details_68_4440.html>;  <https://www.cnbg.com.cn/content/details_68_380.html> | |
| CPL biologicals Pvt. Ltd | <http://cplbio.com/products/>  <http://cplbio.com/press-post/november-17-2016/> | |
| Daiichi Sankyo | [www.daiichisankyo-bt.co.jp/business/index.html#s1](http://www.daiichisankyo-bt.co.jp/business/index.html#s1);  [www.pmda.go.jp/files/000216875.pdf](http://www.pmda.go.jp/files/000216875.pdf);  <https://www.daiichisankyo.com/media_investors/media_relations/press_releases/detail/006842.html> | |
| Dalian Aleph Biomedical Co., Ltd. | [www.alephbio.com/Product/Content.aspx?ProductId=15](http://www.alephbio.com/Product/Content.aspx?ProductId=15)  [www.ncbi.nlm.nih.gov/pmc/articles/PMC4130289/](http://www.ncbi.nlm.nih.gov/pmc/articles/PMC4130289/) | |
| Denka Seiken Co., Ltd. | [www.denka.co.jp/eng/storage/news/pdf/203/20180709_flu_plant_eng.pdf](http://www.denka.co.jp/eng/storage/news/pdf/203/20180709_flu_plant_eng.pdf);  [www.sciencedirect.com/science/article/pii/S2590136219300129](http://www.sciencedirect.com/science/article/pii/S2590136219300129); [www.who.int/immunization/diseases/influenza/Table_clinical_evaluation_influenza_seasonal.xlsx](http://www.who.int/immunization/diseases/influenza/Table_clinical_evaluation_influenza_seasonal.xlsx) | |
| Fluart Innovative Vaccines Kft | <http://fluart.hu/index.php/our-products/> | |
| FORT, ltd. | <http://fort-bt.ru/>;  <https://gmpnews.net/2018/07/fort-increases-its-sales-of-influenza-vaccine/>;  <https://nacimbio.ru/en/vaccine/ultrix/>;  <https://nacimbio.ru/en/vaccine/ultrix-quadri/> | |
| GC Pharma | [www.globalgreencross.com/eng/product/view.do?currentPage=1&idx=11&searchInitial=&searchValue=&searchColumn=&searchColumn2=1002&searchEffect=](http://www.globalgreencross.com/eng/product/view.do?currentPage=1&idx=11&searchInitial=&searchValue=&searchColumn=&searchColumn2=1002&searchEffect=);  [www.globalgreencross.com/eng/product/view.do?currentPage=1&idx=22&searchInitial=&searchValue=&searchColumn=&searchColumn2=1002&searchEffect=](http://www.globalgreencross.com/eng/product/view.do?currentPage=1&idx=22&searchInitial=&searchValue=&searchColumn=&searchColumn2=1002&searchEffect=) | |
| GlaxoSmithKline (GSK) | <https://us.gsk.com/en-us/about-us/vaccines/flu/>;  <https://gsksource.com/pharma/content/dam/GlaxoSmithKline/US/en/Prescribing_Information/Fluarix_Quadrivalent/pdf/FLUARIX-QUADRIVALENT.PDF>;  <https://gskpro.com/en-us/products/flulaval-quadrivalent/>;  <https://www.pharmaceutical-technology.com/projects/gsk-biologicals/>;  <https://ca.gsk.com/media/1511156/gsk-at-a-glance_2018-update-28sept2018v2-final.pdf> | |
| Hualan Biological Engineering Inc. | <http://english.hualanbio.com/products/showproduct.php?lang=en&id=12>;  <http://english.hualanbio.com/products/showproduct.php?lang=en&id=32> | |
| Il-Yang Pharm | [www.ilyang.co.kr/english/product/product01_view.asp?idx=555&page=1&txt_search=&sel_2nd_idx=&sl=&s_gubun=Vaccine](http://www.ilyang.co.kr/english/product/product01_view.asp?idx=555&page=1&txt_search=&sel_2nd_idx=&sl=&s_gubun=Vaccine) | |
| Instituto Butantan | <http://butantan.gov.br/instituto-butantan/about-us>;  <http://butantan.gov.br/instituto-butantan/main-products>;  <https://www.ncbi.nlm.nih.gov/pmc/articles/PMC6069271/> | |
| Institute of Vaccines and Medical Biologicals (IVAC) | [www.path.org/media-center/vietnam-produced-seasonal-influenza-vaccine-licensed-production-and-use/](https://www.path.org/media-center/vietnam-produced-seasonal-influenza-vaccine-licensed-production-and-use/);  <http://ivac.com.vn/san-pham/10/41/ivacflu-s-(vac-xin-cum-mua-dang-manh-bat-hoat)/vien-vac-xin.html>;  <http://ivac.com.vn/uploads/file/IVACFLU-S/HDSD%20CUM%20MUA%20V3%2014-6-19%20nam%20ban%20cau.pdf> | |
| Jiangsu GDK Biotechnology Co., Ltd. | <http://gdkbio.com/productshow.php@cid=3&id=28>;  <https://pubmed.ncbi.nlm.nih.gov/28870140/>;  <https://www.ncbi.nlm.nih.gov/pmc/articles/PMC7423295/> | |
| KM Biologics Co., Ltd. | [www.kmbiologics.com/en/products/vaccines.html](http://www.kmbiologics.com/en/products/vaccines.html);  [www.pmda.go.jp/files/000213511.pdf](http://www.pmda.go.jp/files/000213511.pdf);  [www.nature.com/articles/d42473-018-00213-x](http://www.nature.com/articles/d42473-018-00213-x) | |
| Mechnikov Institute | <https://gmpnews.net/2019/04/launching-of-russian-vaccines-in-latin-america/>;  <http://spbniivs.ru/projects_niivs/> | |
| Microgen | [www.microgen.ru/en/products/vaktsiny/vaktsina-dlya-profilaktiki-grippa-inaktivirovannaya-sovigripp/](http://www.microgen.ru/en/products/vaktsiny/vaktsina-dlya-profilaktiki-grippa-inaktivirovannaya-sovigripp/);  [www.microgen.ru/en/products/vaktsiny/ultravak-vaktsina-grippoznaya-allantoisnaya-zhivaya/](http://www.microgen.ru/en/products/vaktsiny/ultravak-vaktsina-grippoznaya-allantoisnaya-zhivaya/) | |
| Sanofi Pasteur | <https://sanofiflu.com/fluzone-high-dose-influenza-vaccine.html>;  <https://sanofiflu.com/fluzone-quadrivalent-influenza-vaccine.html>;  <https://sanofiflu.com/flublok-quadrivalent-influenza-vaccine.html>;  [www.sanofi.com/en/media-room/press-releases/2017/2017-10-12-07-00-00#:~:text=About%20Sanofi%20Pasteur,)%20and%20Shenzhen%20(China);](http://www.sanofi.com/en/media-room/press-releases/2017/2017-10-12-07-00-00#:~:text=About%20Sanofi%20Pasteur,)%20and%20Shenzhen%20(China);)  [www.biospace.com/article/sanofi-finally-closes-a-deal-pays-750-million-for-this-connecticut-biotech-/](http://www.biospace.com/article/sanofi-finally-closes-a-deal-pays-750-million-for-this-connecticut-biotech-/) | |
| Seqirus | [www.seqirus.com/manufacturing/global-manufacturing-network](http://www.seqirus.com/manufacturing/global-manufacturing-network);  [www.seqirus.com/products](http://www.seqirus.com/products) | |
| Serum Institute of India Pvt. Ltd. | [www.seruminstitute.com/product_influenza_vaccines.php](http://www.seruminstitute.com/product_influenza_vaccines.php);  [www.seruminstitute.com/health_faq_influenza.php](http://www.seruminstitute.com/health_faq_influenza.php);  [www.ciplamed.com/content/nasovac-s-intranasal-spray](http://www.ciplamed.com/content/nasovac-s-intranasal-spray) | |
| Sinovac Biotech Ltd. | [www.sinovac.com/?optionid=459](http://www.sinovac.com/?optionid=459) | |
| SK Bioscience | [www.skbioscience.co.kr/page/en/about02.do](http://www.skbioscience.co.kr/page/en/about02.do);  [www.skbioscience.co.kr/page/en/ourBusiness01.do](http://www.skbioscience.co.kr/page/en/ourBusiness01.do) | |
| SPbNIIVS | <http://spbniivs.ru/product/flu-m/>;  <http://spbniivs.ru/wp-content/uploads/2019/11/flu_m_tetra.pdf> | |
| Takeda | [www.takeda.com/newsroom/newsreleases/2014/takeda-selected-as-recipient-of-supplemental-government-subsidy-in-japan-to-expand-production-capacity-for-cell-culture-pandemic-influenza-vaccine/](http://www.takeda.com/newsroom/newsreleases/2014/takeda-selected-as-recipient-of-supplemental-government-subsidy-in-japan-to-expand-production-capacity-for-cell-culture-pandemic-influenza-vaccine/);  [www.takeda.com/newsroom/newsreleases/2014/takeda-announces-the-new-drug-application-approval-of-cell-cultured--influenza-vaccine-h5n1-takeda/](http://www.takeda.com/newsroom/newsreleases/2014/takeda-announces-the-new-drug-application-approval-of-cell-cultured--influenza-vaccine-h5n1-takeda/) | |
| Zydus Cadila | <https://zyduscadila.com/research>;  <https://zyduscadila.com/public/pdf/pressrelease/Zydus-Cadila-launching-Tetravalent-Influenza-vaccine.pdf>;  [www.hindustantimes.com/business/cadila-gets-nod-for-swine-flu-vaccine-launch/story-2erkQKbXiGJyzJtro79HkM.html](https://www.hindustantimes.com/business/cadila-gets-nod-for-swine-flu-vaccine-launch/story-2erkQKbXiGJyzJtro79HkM.html) | |
